# Optical characterization of In-flushed InAs/GaAs quantum dots emitting a broadband spectrum with multiple peaks at ~1 μm

**DOI:** 10.1186/s11671-015-0941-0

**Published:** 2015-05-27

**Authors:** Shigehiro Kitamura, Masaya Senshu, Toshio Katsuyama, Yuji Hino, Nobuhiko Ozaki, Shunsuke Ohkouchi, Yoshimasa Sugimoto, Richard A Hogg

**Affiliations:** Graduate School of Engineering, University of Fukui, Fukui, 910-8507 Japan; Faculty of Systems Engineering, Wakayama University, Wakayama, 640-8510 Japan; NEC Corporation, Tsukuba, Ibaraki 305-8501 Japan; National Institute for Materials Science (NIMS), Tsukuba, Ibaraki 305-0047 Japan; Department Electronic and Electrical Eng, University of Sheffield, Sheffield, S3 7HQ UK

**Keywords:** Quantum dot, In-flush, MBE, Time-resolved PL, OCT, Broadband light source

## Abstract

We investigated optical properties of In-flushed InAs quantum dots (QDs) grown on a GaAs substrate by molecular beam epitaxy. By using the In-flush technique for setting the height of self-assembled InAs QDs, we have tuned the emission wavelength of InAs QDs to the ~1 μm regime, which can be utilized as a non-invasive and deeply penetrative probe for biological and medical imaging systems. The controlled emission exhibited a broadband spectrum comprising multiple peaks with an interval of approximately 30 meV. We examined the origin of the multiple peaks using spectral and time-resolved photoluminescence, and concluded that it is attributed to monolayer step fluctuations in the height of the In-flushed QDs. This feature can be advantageous for realizing a broadband light source centered at the ~1 μm regime, which is especially suitable for the non-invasive cross-sectional biological and medical imaging system known as optical coherence tomography.

## Background

Optical biological/medical imaging system that uses near-infrared (NIR) light has been developed as a non-invasive and deeply penetrative diagnosis method. In particular, the NIR light of ~1 μm wavelength has a relatively long penetration depth in living aqueous tissues, since the absorption of light by the main ingredients of tissue, oxyhemoglobin (HbO_2_) and water (H_2_O), is minimized at 1.05 μm [1]. Among the imaging systems, optical coherence tomography (OCT) [2] has been extensively developed and widespread in various medical fields over the last few decades [3]. OCT is a non-invasive cross-sectional imaging system based on a low-coherence interferometer. The axial resolution of OCT is governed by the center wavelength and bandwidth of the light source, e.g., the axial resolution given by the expression 0.44 × *λ*_0_^2^/Δ*λ* for a light with a Gaussian spectral shape centered at *λ*_0_ and a bandwidth of Δ*λ* [4]. Thus, the development of a broadband light source with a 1-μm-centered wavelength is crucial for obtaining OCT images with a high-resolution and a large penetration depth in biological and medical samples. For instance, light with a bandwidth of 100 nm centered at an emission wavelength of 1.05 μm can be expected to have an axial resolution of 4.85 μm, which is comparable with cell sizes in biological samples.

So far, we have developed the NIR broadband light source based on self-assembled InAs quantum dots (QDs) for OCT light source [5–7]. The InAs QDs are derived from strain induced by a lattice mismatch between the deposited InAs and the GaAs substrate, and the ensemble of QDs has distributions of size and composition. Thus, the ensemble of InAs QDs emits a broadband spectrum because of inhomogeneous broadening and has been recognized as a good material for OCT light sources [8]. Recently, a broadband light source based on InAs QDs was developed and achieved a bandwidth of over 200 nm [9–12]. However, the InAs/GaAs QDs typically emit light of approximately 1.2–1.3 μm wavelength; therefore, we have developed various methods to control the emission wavelength of InAs QDs [13]. For the adjustment of the emission wavelength to the 1 μm regime, we have utilized the In-flush technique [14, 15]. The In-flush technique was previously developed for setting the QD height to a constant value and obtaining a highly homogeneous QD emission wavelength [16]. This method can also be applied to the control of the InAs QD emission wavelength and enables the creation of a broadband light source by combining the emission–wavelength-controlled QDs [14, 15, 17–19]. In our previous work regarding the control of the emission wavelength via the In-flush method, we have found that the In-flushed QDs exhibit multiple emission peaks in the emission spectrum at the ~1 μm regime [14]. This result implies a unique optical feature of the In-flushed QDs, which differ from the conventional InAs QDs, and the emission could be suitable for a broadband light source emitting at the 1 μm regime. In this work, we investigate the origin of the multiple emission peaks of the In-flushed QDs through spectral and temporal photoluminescence (PL) measurements, and discuss the potential of the In-flushed QDs for applications to the OCT light source.

## Methods

**Fig. 1:**
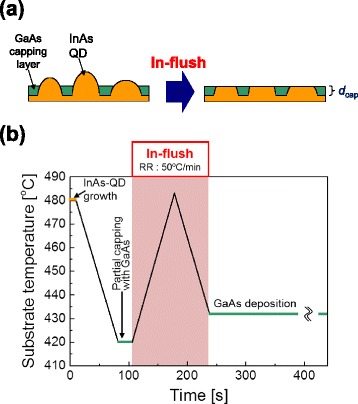
**a** Schematic drawing of the In-flush process. **b** Growth scheme of the In-flush process

The samples were grown on a GaAs substrate by molecular beam epitaxy (MBE). As schematically shown in Fig. 1a, In-flushed QDs were formed by annealing InAs QDs partially capped with a GaAs layer. Self-assembled InAs QDs were grown on a GaAs substrate with the deposition of two monolayers (MLs) of InAs by the conventional strain-induced Stranski–Krastanov (S–K) growth mode [20]. The growth temperature of the QD was approximately 480 °C. The density of the grown QDs was approximately 4.6 × 10^10^ cm^−2^, and the average size was as follows: approximately 48 nm in base diameter and 4.5 nm in height. After the growth of the QDs, the substrate temperature was decreased to 420 °C and a GaAs capping layer with a specific thickness (*d*_cap_) was deposited onto the QDs. Subsequently, the substrate temperature was rapidly increased to approximately 480 °C and decreased to approximately 430 °C (In-flush) to eliminate the excess QDs over the capping layer, resulting in QDs with a height controlled by *d*_cap_. The ramp rate of the increasing/decreasing temperature during the In-flush was approximately 50 °C/min. We prepared In-flushed QDs with a *d*_cap_ of 2.0 nm for the adjustment of the emission wavelength to ~1.05 μm. The In-flushed QDs were embedded between 30-nm-thick GaAs spacer layers and 10-nm-thick Al_0.3_Ga_0.7_As barrier layers for the confinement of free (electron and hole) carriers. The growth scheme of the In-flush is shown in Fig. 1b. Throughout the QD growth processes, including the In-flush process, an arsenic flux of 2 × 10^5^ Torr was continuously irradiated onto the sample. We also prepared a reference sample (as-grown QDs) embedding as-grown InAs QDs formed without the partial capping and annealing processes instead of embedding the In-flushed QDs.

**Fig. 2:**
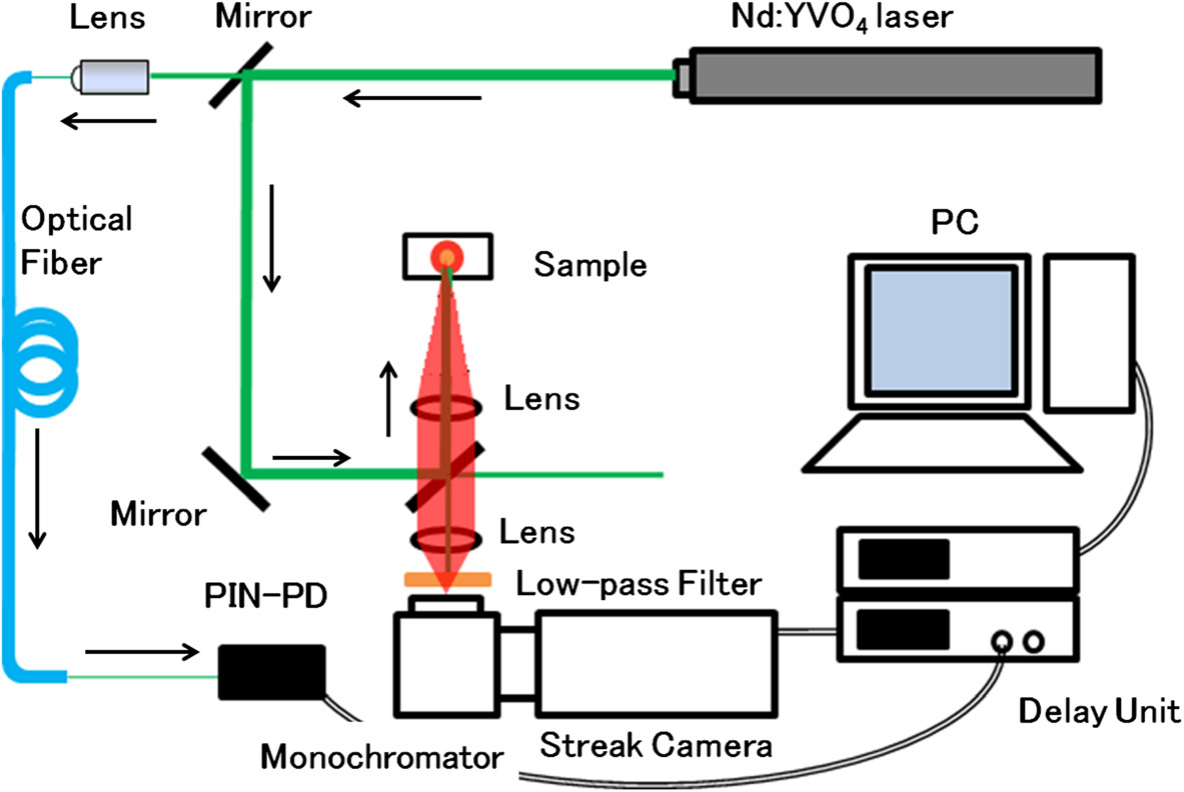
Schematic image of the time-resolved PL measurement system

The optical properties of the In-flushed QDs sample were investigated using PL measurements at room temperature (RT) and time-resolved PL measurements at liquid nitrogen temperature (LNT). For the RT-PL measurements, a focused He–Ne (*λ* = 632.8 nm) laser was employed for photoexcitation. The excitation power dependence of the PL spectra was investigated using the variation of the power density of the excitation laser spanning approximately 100–300 W/cm^2^. The time-resolved PL was measured with a streak camera combined with an Nd:YVO_4_ mode-locked laser (emission wavelength: 532 nm, pulse duration: 6.3 ps, repetition rate: 1 MHz) at LNT, as shown in Fig. 2.

## Results and discussion

**Fig. 3:**
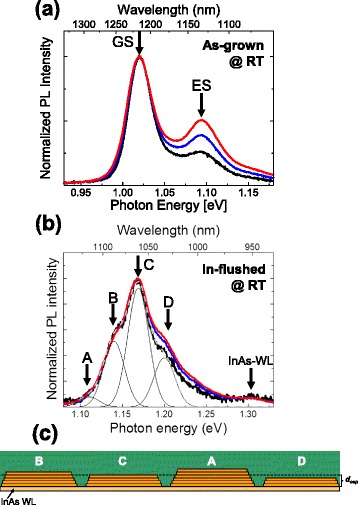
Photoexcitation power dependence of RT–PL obtained from **a** as-grown InAs QDs and **b** In-flushed QDs. The excitation power densities were approximately 100 (*black*), 200 (*blue*), and 300 (*red*) W/cm^2^. **c** Schematic drawing of ML-step height fluctuations of the In-flushed QDs

Figure 3a, b shows the RT–PL spectra obtained using various photoexcitation powers (approximately 100, 200, and 300 W/cm^2^) from the as-grown QDs and the In-flushed QDs, respectively. As shown in Fig. 3a, the as-grown QDs emit a dominant peak at approximately 1.02 eV (*λ* = 1.22 μm) and an additional peak at approximately 1.09 eV (*λ* = 1.13 μm). These emission peaks originate from discrete states due to the quantum confinement of electrons and holes in QDs; the lower energy emission arises from the recombination between the fundamental ground states (GS) of electrons and holes, and the higher energy emission arises from the recombination between excited states (ES). The inhomogeneous broadening of the peaks originate from the size and composition distributions of the QDs. The full width at half maximum (FWHM) of the GS emission is approximately 36 meV. As seen in the normalized PL spectra, the different excitation power dependence between the GS and ES emissions is attributed to the state filling of the GS with photogenerated carriers, resulting in a saturation of GS emission intensity and a subsequent increase in ES emission intensity.

On the other hand, the In-flushed QDs emit a PL spectrum centered at approximately 1.17 eV (*λ* = 1.06 μm) with multiple peaks, which can be fitted with a Gaussian function centered at 1.11, 1.14, 1.17, and 1.20 eV with a FWHM of 29 meV, as labeled A–D in Fig. 3b. The fitting line is indicated by a gray dashed line. The weak emission peak at approximately 1.31 eV (*λ* = 0.950 μm) is due to the InAs wetting layer (WL). The emission wavelength, blue-shifted to the 1.05 μm regime, indicates the controllability of the In-flush method. The multiple peaks with intervals of approximately 30 meV, which were also seen in our previous report [14], can be attributed to the step-like height fluctuation of the In-flushed QDs. As schematically shown in Fig. 3c, the height of the In-flushed QDs decreases to *d*_cap_, which is approximately 2 nm (6 ML), and they have a truncated pyramid (disk-like) shape. The decrease in the ratio of the height to base diameter (approximately 48 nm) should result in a change in the quantum confinement of electrons and holes from a three-dimensional confinement to a quasi-one-dimensional strong confinement in the height direction. This results in a large change in the bandgap energy of the In-flushed QDs with a variation of even 1 ML in height. Thus, the emission peak energy should be divided with step values corresponding to the ML-step height. Bimberg et al. have also reported the similar multi-peak emission due to the ML-step height fluctuation in a shell-like formation of InAs QDs by the metal–organic chemical vapor deposition method [21, 22]. They reported the interval of emission energy for one ML of the truncated QD as 29–51 meV and the energy shift between the truncated QDs with heights of 6–7 ML as 32 meV. This value is close to our observed interval energy between the peaks. Moreover, the reported FWHM of the peak was 29 meV, which is almost the same as our measured value. The decrease in the FWHM from that of as-grown QDs implies that the transformation of the QD structure to a quasi-one-dimensional confinement structure resulted in a reduction of the inhomogeneous broadening. The remaining inhomogeneous broadening may be due to the distribution of In compositions and the base diameters of the QDs, causing lateral quantum confinement.

**Fig. 4:**
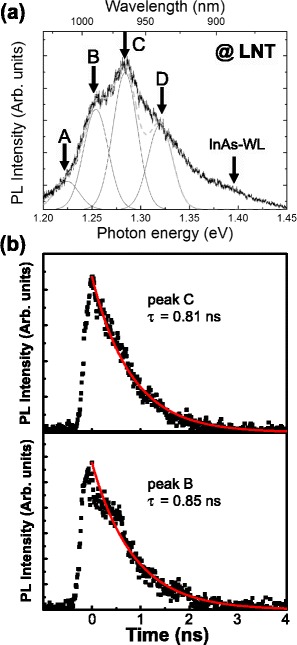
**a** LNT PL spectrum obtained from the In-flushed QDs. **b** PL time decay measured at peaks B and C shown in **a**

As seen in the RT–PL (Fig. 3b), normalized emission peaks A–C (1.11, 1.14, and 1.17 eV) exhibit identical intensities as a function of the excitation power, whereas the emission intensity spanning 1.18–1.26 eV increases slightly. This suggests that emission peaks A–C originate from the GS emissions of the In-flushed QDs with different heights, and the emissions at 1.18–1.26 eV include the ES emissions of the QDs in addition to the GS emissions of QDs at 1.20 eV (peak D). The difference in the emission line compared to the fitting line can be attributed to the additional ES emissions. In order to confirm this scenario, we measured the time-resolved PL for the In-flushed QD sample and verified the origins of the emissions. Figure 4a shows a PL spectrum obtained from the In-flushed QDs at LNT. This spectrum can also be fitted with multiple Gaussian peaks A–C (1.22, 1.25, and 1.28 eV) and broad emissions spanning 1.30–1.35 and 1.35–1.42 eV. These can originate from the corresponding emission centers seen in the RT–PL spectrum, whose energy values shift with the temperature decrease. In the broad emission spanning 1.30–1.35 eV, the ES emissions and the GS emission that peaks at approximately 1.32 eV (peak D) are included. The difference between the emission line and the fitting line (dashed gray line) could be due to the additional ES emissions. The emission spanning 1.35–1.42 eV, which is centered at approximately 1.39 eV, should be from InAs WL. We first measure the PL time evolution of apparent peaks B and C. As shown in Fig. 4b, the photoluminescence intensity initially rises and exhibits a peak at approximately 300 ps after the excitation and then falls with a single exponential decay. The time scale is indicated with the intensity peak time as zero. The rise time can be understood as the time for the creation of photoexcited carriers in the GaAs layer and the capture of these carriers by QDs through InAs WL. In addition, in the short-time range (~0.6 ns), there is a slight fluctuation in the PL decay, which is especially obvious in peak B. This could be influenced by the relaxation process from the InAs WL to the GS state through the ES states of the QD, as discussed in the next paragraph. However, the main decays of peaks B and C can be fitted with a single exponential curve, and the decay times are estimated to be 0.85 and 0.81 ns, respectively. When considering that the typical decay time of the emission from the GS of InAs/GaAs QDs is ~1 ns [23–26], these results demonstrate that the peaks originate from the GS of QDs with a different height of one ML, as was previously discussed regarding the RT–PL result.

**Fig. 5:**
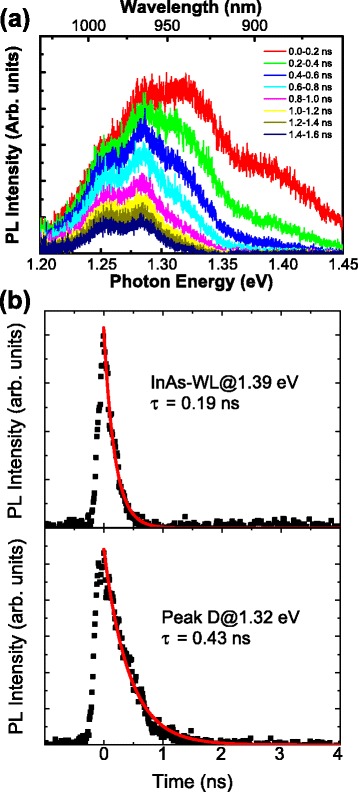
**a** Time-resolved PL spectrum obtained from the In-flushed QDs at LNT. **b** PL time decay measured at 1.39 and 1.32 eV in the spectrum shown in **a**

Next, we examined the PL time evolution at the higher energies spanning 1.30–1.35 and 1.35–1.42 eV. To clarify the time evolution of the entire higher energy range, we obtained time-resolved PL spectra with time intervals of 0.2 ns, as shown in Fig. 5a. The origin of the timescale is set at the peak time of the GS emission shown in Fig. 4b. The PL spectra exhibit the fastest decay in the emission spanning 1.35–1.42 eV and a subsequent fast decay in the range of 1.30–1.35 eV. Figure 5b shows the decay time curves obtained at 1.39 and 1.32 eV. The decay times estimated by single exponential fitting are 0.19 and 0.43 ns, respectively. By considering that the emission at 1.39 eV originates from the InAs WL, we can assume that the fast decay of 0.19 ns is mainly due to the relaxation of captured photoexcited carriers by the InAs WL into the ES of the QDs. The subsequent relaxation of the carriers into the GS of the QDs occurs with the time constant of 0.43 ns. This sequential relaxation of photoexcited carriers with an increase in time constant has been also observed in the normal self-assembled InAs/GaAs QDs [23–26]. Thus, the time-resolved PL measurements indicate that there is no significant difference between the In-flushed QD and the normal InAs QD; the photoexcited carriers in a GaAs layer follow the typical relaxation ladder dynamics to the GS of the QDs via the InAs WL and the ES.

From the aforementioned spectral and time-resolved PL results, we conclude that the multiple emission peaks that appear in the PL spectra obtained from the In-flushed QDs originate from multiple emission centers with ML-step fluctuations of the height of the truncated QDs. These multiple centers result in emission broadening and are useful for realizing a broadband light source, which is especially applicable to the OCT. The bandwidth of the In-flushed QDs is approximately 75 nm with contributions from only the GS emissions and increases up to 100 nm with the addition of ES emissions. This indicates that an axial resolution that is less than 5 μm in air can be achieved using this as a light source in OCT system. Furthermore, precise control of the emission wavelength can be executed by the variation of *d*_cap_, as previously reported [14, 15], and further broadening and control of the center wavelength can be expected. Thus, the In-flushed QDs offer a novel approach for developing a broadband light source centered at 1.05 μm. In addition, this approach using the In-flushed method might be effective for other In-incorporated QD systems.

## Conclusions

The optical properties of In-flushed QDs have been investigated using spectral and time-resolved PL measurements. The RT–PL spectrum shows multi-peak emissions from the In-flushed QDs. The emission energy intervals and the excitation power dependence of the multi-peak emissions indicate that the peaks originate from the GS emissions of In-flushed QDs with ML-step height fluctuation. The time-resolved PL measurements also demonstrate that the multi-peak emissions have almost identical decay times and are similar to the typical decay time of the GS of QDs. This feature of the In-flushed QDs can be suitable for broadband emission centered at the ~1 μm range.
